# Supplementary material for: Rapid turnover of life-cycle-related genes in the brown algae
Source: Genome Biol. 2019 Feb 14;20:35. doi: 10.1186/s13059-019-1630-6 (PMC6374913; doi:10.1186/s13059-019-1630-6)
Supplement: Supplementary file 3 — Protein alignments, corresponding Gblocks html file and CDS sequences for the lineage conserved gametophyte-biased genes. (ZIP 2109 kb) [file 13059_2019_1630_MOESM3_ESM.zip › Con_GA_alignments/Ec-26_002470.aa_ali.fasta-gb.htm]

Ec-26\_002470.aa.fasta.trans.aa\_ali.fasta


## Gblocks 0.91b Results

Processed file: **Ec-26\_002470.aa.fasta.trans.aa\_ali.fasta**  
Number of sequences: **2**  
Alignment assumed to be: **Protein**  
New number of positions: **649** (selected positions are underlined in blue)

```
                         10        20        30        40        50        60
                 =========+=========+=========+=========+=========+=========+
Ec-26_002470     ---------MSAHG---RRRSSALAAQVLEAIRETDEEIFEEILQYDLVALGGGVAAGYW
MSTRG.40613.1    MDLNGTIDAMSAPDYGSRRRSSMMAAQVLEAIRETDEEIYEETFQYDLVALGGGVATGYW
                                  ###########################################


                         70        80        90       100       110       120
                 =========+=========+=========+=========+=========+=========+
Ec-26_002470     GEAIAGSLGENDYTAAIVSGDPAGFAPFERPALSKTCLNPGAKGSRDFSCSSSSDFPFVC
MSTRG.40613.1    GEAIVRYLRKQRRTAAIVSGDPTGFAPFERPALSKECLNPASSKTRQLSGGLPS-FPFIC
                 ###################################################### #####


                        130       140       150       160       170       180
                 =========+=========+=========+=========+=========+=========+
Ec-26_002470     QGSGGEAHDPEWYKTNGITILSRCQAVSVDLDQKFLKVKRVTA-SNGELVFGDTIKVVYG
MSTRG.40613.1    NGSGGEPHDPEWYRANGITILSSCQAMYVDLDHKFLKVKRFTAGCDGELVVGDTIKVVYG
                 ###########################################   ##############


                        190       200       210       220       230       240
                 =========+=========+=========+=========+=========+=========+
Ec-26_002470     RLLIATGARPRKIADEIKSLDNISKGLQGSCCCTRHPDVDGSPLACTFPDRFGFGSVHYL
MSTRG.40613.1    RLMIATGARARRIAERLGPPENISRGLQGSCCCTRHPDVGGSPLACTFPDRFGFGSVHYM
                 ############################################################


                        250       260       270       280       290       300
                 =========+=========+=========+=========+=========+=========+
Ec-26_002470     RDFGDTVKLVHAMGRVEADGQDTCKEPVVLIGGGFISCEVAAAVATHCPGMQLVMVMPGE
MSTRG.40613.1    RDFGDTVKLVHAMGRVEADGQDTCKEPVVLLGGGFISCEVAAAIATHCPGMQLVMVLPKD
                 ############################################################


                        310       320       330       340       350       360
                 =========+=========+=========+=========+=========+=========+
Ec-26_002470     DVMSSSGFGKEVCHFYEKQLARVGVAFAKGYRVNRLWGIEEEGCFPTLERSLQGSSMKKT
MSTRG.40613.1    DVMSSSGFGKDVCHFYEKQLARVGVAFAKGYRVERLWGIEEEGNFPTLERSLQGSSMKKT
                 ############################################################


                        370       380       390       400       410       420
                 =========+=========+=========+=========+=========+=========+
Ec-26_002470     FPRTFGPADPHFTDCRGVVLSNTQTGEKVWLLARFVVVGIGSVPNSDLFLKSLVLSEDGG
MSTRG.40613.1    FPRTFGPADPHFTDCRGVVLSHRQNGEQVWVAARFVVVGIGSIPNSELFRHSLVLSEDGG
                 ############################################################


                        430       440       450       460       470       480
                 =========+=========+=========+=========+=========+=========+
Ec-26_002470     IITDSYLRTSHPSGDVFAAGDVACVPVTLGGNGDTSFMAIANRSEHVKAARDMGTFVAGM
MSTRG.40613.1    IVTDSSLRTSHPSGDVFAAGDVACAPVPLRGNGEGNFMATAIRSEHVKSARDMGTFAAAM
                 ############################################################


                        490       500       510       520       530       540
                 =========+=========+=========+=========+=========+=========+
Ec-26_002470     MLGGDDSGESYNPVPHMYSRFLDISWKFYGFARGEVCVLGMETFPMTRTFGAFWLHGGKI
MSTRG.40613.1    MLAGSDTGESYNPVPHVYSRFLDISWKFYGFVRGEVCVLGMENFSVTRTFGAFWLDGGKI
                 ############################################################


                        550       560       570       580       590       600
                 =========+=========+=========+=========+=========+=========+
Ec-26_002470     VGAFLEEDPSSGVDHSAILERMARERPAVLSIKRLRKCPLEKLLDNPHCLEPPKLGVGEF
MSTRG.40613.1    VGAFLEEDPASGVDHSAILERMAREQPPVLSIKRLRKCPLKELLANPYCLAPPKLGVGEF
                 ############################################################


                        610       620       630       640       650       660
                 =========+=========+=========+=========+=========+=========+
Ec-26_002470     HADTDETSIEEAFRKRAGGASTAKRADMASIMQELGADWDEDELEDALRALDPSDSGSVN
MSTRG.40613.1    HADTDETSVEDSFRKYAGANAKVKRADMAAVMQELGADWDNNEVEDALGALDPSGSDSVH
                 ############################################################


                        670
                 =========+
Ec-26_002470     FSDFVEWWSN
MSTRG.40613.1    LSAFVKWWSN
                 ##########
```

```
Parameters used
Minimum Number Of Sequences For A Conserved Position: 2
Minimum Number Of Sequences For A Flanking Position: 2
Maximum Number Of Contiguous Nonconserved Positions: 8
Minimum Length Of A Block: 10
Allowed Gap Positions: None
Use Similarity Matrices: Yes
```

```
Flank positions of the 3 selected block(s)
Flanks: [18  114]  [116  163]  [167  670]  

New number of positions in Ec-26_002470.aa.fasta.trans.aa_ali.fasta-gb:  649  (96% of the original 670 positions)
```
